# The Utility of Psychological Capital Theory in Rural Adolescent Mental Health Research

**DOI:** 10.1177/13591045261434614

**Published:** 2026-03-13

**Authors:** Angela J. Preston, Lynn Rew, Cara C. Young, John Lowe, Huaxin Song

**Affiliations:** 1 School of Nursing, The University of Texas, Austin, TX, USA; 2 School of Nursing, 12347 The University of Texas at Tyler, Tyler, TX, USA

**Keywords:** youth, stigma, rural mental health, theory, hope, self-efficacy, resilience, optimism, wellbeing, anxiety

## Abstract

**Background:**

Very few studies have been conducted to explore psychological capital (combined hope, self-efficacy, resilience, and optimism known as PsyCap) and mental health among rural U.S. adolescents.

**Purpose:**

The purpose of this study was to explore the relationship between PsyCap and mental health using a health equity lens.

**Methods:**

A sample of 59 rural U.S. adolescents completed the survey packet. Descriptive and inferential statistics were conducted on the RStudio platform.

**Findings:**

PsyCap was significantly associated with wellbeing and anxiety. Non-binary adolescents reported significantly lower PsyCap. While the overall sample reported perceived rejection above the national norm, non-binary adolescents reported the highest levels.

**Discussion:**

Psychological capital theory served as a useful framework for exploring mental health among rural adolescents. More research needs to be conducted with large inclusive samples of rural adolescents who live on the margins. Examination of mental health services in rural settings should be considered.

## Plain Language Summary

Psychological Capital Theory, along with aspects of Minority Stress Theory, is useful for exploring mental health among rural adolescents.

• Higher psychological capital is associated with stronger in-group identity among rural adolescents.

• Non-binary adolescents report significantly higher perceived rejection and lower psychological capital than male and female adolescents.

• Psychological capital is significantly associated with mental health among rural adolescents.

• Rural adolescents report higher levels of perceived rejection than the general population.

## Introduction

Adolescents living in rural, underserved communities are at heightened risk for mental health problems (Centers for Disease Control and Prevention [CDC], 2021; Nehme et al., 2021). Complex factors contribute to health disparities, including lack of access to qualified healthcare providers (Centers for Disease Control and Prevention [CDC], 2021), inequitable distribution of healthcare resources (Scott et al., 2009), and perceived rejection and stigma among those living on the margins and outside of social norms (Carpenter-Song & Snell-Rood, 2017; National Institutes of Mental Health and Health Disparities [NIMHD], 2023; Turner & Young, 2007). Stigma and rejection are related to suicidal ideation (Cheek et al., 2021; Wolke & Lereya, 2015), and rural adolescents encounter twice the risk of suicide as urban peers (National Institutes of Health, 2020). Furthermore, adolescents experience rejection and stigma related to conflicting identities such as minority status, gender, and social norms (Meyer, 2003; NIMHD, 2023).

Development of inner psychological strengths, an asset available to all adolescents, offers an opportunity to address national priorities in mental health promotion (Kann et al., 2018; US Department of Health and Human Services [USDHHS], n. d.). Psychological strengths, based on the positive psychology paradigm, have gained traction in mental health promotion because they are state-like characteristics which can be trained through positive mindset exercises (Preston, 2024). Psychological capital (PsyCap) is a theoretical combination of psychological strengths amenable to development and intervention (Luthans et al., 2007; Song et al., 2019).

### Psychological Capital and Minority Stress Theory

Psychological capital is when an individual possesses a combination of the state-like characteristics hope, self-efficacy, resilience and optimism (referred to as *the HERO within*). PsyCap Theory predicts that this particular combination of attributes has a significantly stronger impact on performance and wellbeing than any individual attribute alone (Luthans et al., 2007). PsyCap and positive outcomes in work performance, academic success, and wellbeing are well-established among international samples of university students and adults (Avey et al., 2010).

Far fewer studies have been conducted to explore the relevance of PsyCap Theory in relation to adolescent mental health. Therefore, in this preliminary study, we tested the applicability and utility of PsyCap Theory as an avenue for mental health promotion among rural adolescents in the southern U.S. To better understand socioenvironmental factors which may impact underserved rural adolescents, we also used aspects of Minority Stress Theory to guide the methods undertaken. Minority Stress Theory states that when a conflict occurs between one’s self-identity and the identity that said person believes others to have of them, increased anxiety may occur, as well as reduced wellbeing (Meyer, 2003). Minority Stress Theory suggests socioenvironmental factors may impact how one perceives rejection from others in relation to their identity. When there is social labeling, negative stereotyping, distinct categorizing or separation, and power asymmetry resulting in exclusion or rejection between an individual and the majority of others (due to individual personal characteristics), this may be referred to as stigma (Andersen et al., 2022; Link & Phelan, 2001).

A very small rural high school for this pilot study was selected, because adolescents from very small rural communities in particular are known to be left out of research, while at the same time they do not have the level of diversity as larger urban areas and certain groups are categorized based on particular undesirable characteristics, labeled, and isolated, and notably, these isolated rural groups of adolescents tend to compose a larger percentage of adolescent suicides (Alston et al., 2022). Those who don’t look-like or relate to the greater majority of the student body (or teachers at the school) are placed at heightened risk of perceiving themselves to not fit in and feel rejection from others (e.g, lesser sense of in-group identity and rejection), even if they internally see themselves as equally-capable students and citizens of the rural community.

In this study, we tested the associations between PsyCap and mental health outcomes among this group of underserved rural adolescents using our conceptual framework based on aspects of PsyCap Theory and Minority Stress Theory. We collected primary data and took into account socioenvironmental factors such as race, gender, and poverty, in the linear regression model. Lastly, the developmental period of adolescence is a key transitional period for identity-development, so we explored how self-reported identities (i.e., racial or gender identity, in-group identity) correlated with state-like PsyCap and perceived rejection.

To our knowledge, no studies have been conducted as of yet to explore the relationship between PsyCap, perceived rejection, and mental health among adolescents. This pilot study provides preliminary evidence that PsyCap Theory is relevant for researchers seeking to promote health equity and positive adolescent development by accounting for socioenvironmental characteristics (see Figure 1). The aims of this study were: (1) to explore the relationship between PsyCap and perceived rejection; (2) to examine differences in PsyCap and perceived rejection between self-identified groups of adolescents; and (3) to investigate relationships among PsyCap, anxiety, and subjective wellbeing (operationalized through measures of life satisfaction, meaning and purpose, and positive affect). The associated research questions were.

1. What is the relationship between psychological capital and perceived rejection among rural adolescents?
2. What differences in psychological capital and perceived rejection are there between groups of rural adolescents?
3. What is the relationship between psychological capital and mental health outcomes among rural groups of adolescents?

**Figure 1.**
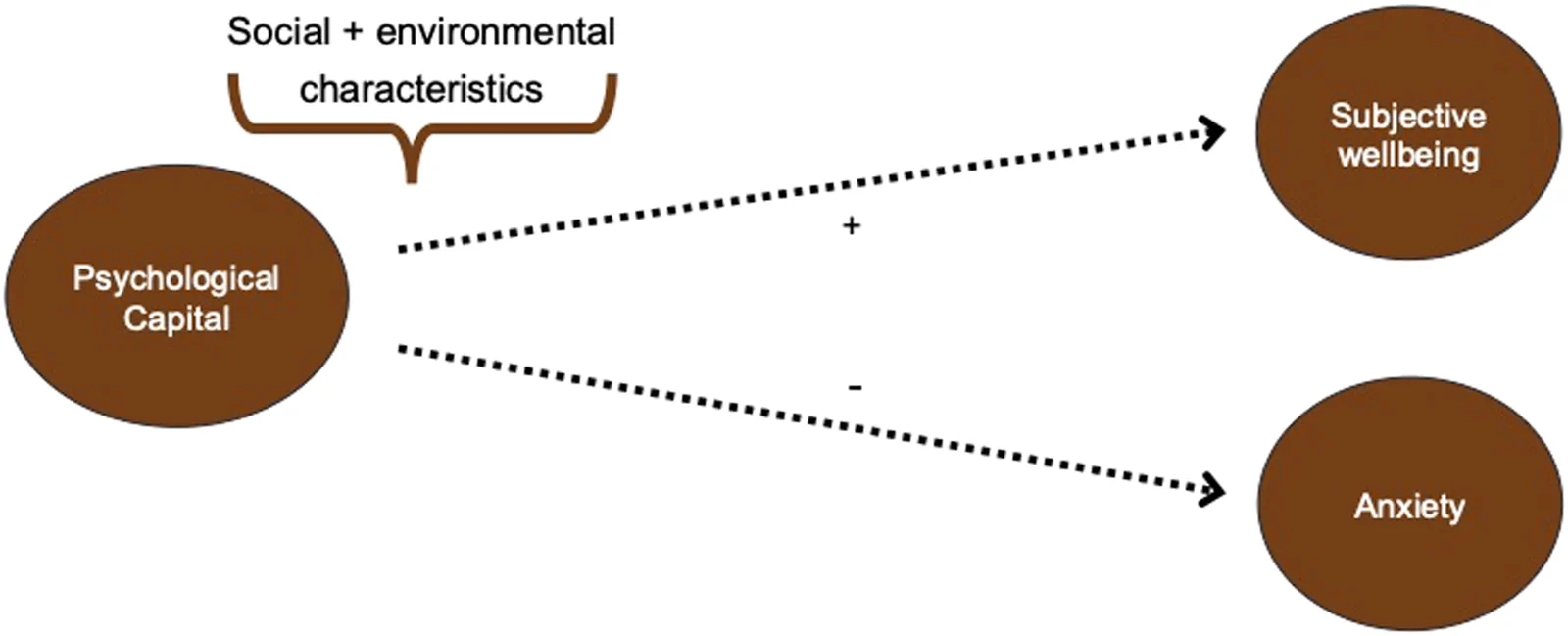
Conceptual study framework based on aspects of psychological capital theory and minority stress theory. *Note.* Subjective wellbeing includes domains of life satisfaction, positive affect, and meaning/purpose. Social + environmental characteristics may include: Gender, race, poverty status, perceived rejection

## Methods

The cross-sectional, correlation study was conducted following approval from the University of Texas at Austin Institutional Review Board. The consent process included parent opt-out consent (declination of consent) and verbal youth assent, modeled in accordance with recommendations made the by CDC for the administration of the Youth Risk Behavior Surveillance Survey System (YRBSS; CDC, 2022).

### Setting and Sample

Participants were recruited from a 2-A high school in rural Texas (e.g., high school enrollment is between 105 - 253 students; University Interscholastic League [UIL], 2025), comprised of 57.8% male and 69.6% white students (TEA, 2022). The primary investigator (PI) met with high school administrators to explain the study purpose. A letter was sent home with study information for all parents to be able to read about and decline student participation in the study in both paper and electronic formats. With the help of the high school principal, the QR link to access the Qualtrics survey packet was emailed to all study hall teachers. The 9^th^-12^th^ grade study hall teachers displayed the QR link on the classroom white board during study hall period. Students who chose to participate used their personal electronic devices and the survey packet was written in English. Students were not asked questions on sexual orientation at the request of the school district.

### Measures

The following measures were selected to answer the specified research questions based on the conceptual framework guiding this study (see Figure 1).

#### Investigator-Developed Demographic Form

A 9-item investigator-developed demographic form asked questions related to age, grade, race, ethnicity, gender, primary caregiver, adoption/foster history, and disability status.

#### In-Group Identity

The Multigroup Ethnic Identity Measure-Revised (MEIM-R) is a 6-item instrument with a 5-point Likert rating system, with a higher score indicating a higher level of in-group identity. A sample item is “I have a strong sense of belonging to my own ethnic group.” When previously used among a sample of rural U.S. youth, the Cronbach’s alpha value was 0.88 (Rew et al., 2014). In this study the Cronbach’s alpha = 0.92.

#### Psychological Capital

The Positive Psychological Capital Questionnaire- English (PPQ-E) is a modified 22-item instrument with a 7-point Likert rating system, with a higher score indicating a higher level of psychological capital. A sample question is “I am committed to doing what it will take to reach my goals” (Preston et al., 2025). When used among a sample of 425 rural adolescents, the Cronbach alpha value was 0.89 (Preston et al., 2023). In this study the Cronbach’s alpha of the total scale = 0.87 and values for each of the subscales was .89 (hope), .83 (self-efficacy), .75 (resilience), and .72 (optimism).

#### Perceived Rejection

The NIH Toolbox® Pediatric Item Bank v2.0 - Perceived Rejection (Ages 8–17) – Fixed Form was used to operationalize the socioenvironmental experience of rejection based on our guiding theoretical framework (HealthMeasures, 2025; Meyer, 2003). It is a 5-item instrument with a 5-point Likert rating system, with higher scores indicating higher levels of perceived rejection. A sample question is “Describe how often people in your life make you feel like you don’t fit in.” When used among a sample of 1038 U.S. youth the Cronbach alpha was 0.90 (Dietz et al., 2022). In this study the Cronbach’s alpha = 0.87.

#### Life Satisfaction

The PROMIS Pediatric Item Bank v1.0 - Life Satisfaction – Short Form 8a was used to operationalize one aspect of subjective wellbeing. It is an 8-item instrument with a 5-point Likert rating system, with higher scores indicating higher levels of life satisfaction. A sample question is “I am satisfied with my life.” When used among a sample of 2,988 children (ages 5–17), the Cronbach alpha was 0.89 (Forrest et al., 2018). In this study the Cronbach’s alpha = 0.95.

#### Meaning and Purpose

The PROMIS Pediatric Item Bank v1.0 - Meaning and Purpose – Short Form 8a is an 8-item instrument with a 5-point Likert rating system, with higher scores indicating higher levels of meaning and purpose. A sample question is “I have a reason for living.” When used among a sample of 2,885 children (ages 5–17), the Cronbach alpha value was 0.90 (Forrest et al., 2019). In this study the Cronbach’s alpha = 0.93.

#### Positive Affect

The NIH Toolbox Item Bank v2.0 – Positive Affect (Ages 13-17) – Fixed Form is a 10-item instrument with a 5-point Likert rating system, with higher scores indicating higher levels of positive affect. A sample question is “In the past 7 days, I felt peaceful.” When used among a sample of 1,004 youth, the coefficient alpha value was 0.90 (Forrest et al., 2018). In this study the Cronbach’s alpha = 0.94.

#### Anxiety

The Neuro – QOL Item Bank v1.0 - Pediatric Anxiety – Short Form is an 8-item instrument with a 5-point Likert rating system, with higher scores indicating higher levels of anxiety. A sample question is “In the past 7 days, I felt worried.” When used among a sample of pediatric chemotherapy patients, the Cronbach alpha value was 0.80 (Hinds et al., 2015). In this study the Cronbach’s alpha = 0.87.

### Data Analysis

Data were reviewed for missing responses, violation of assumptions, and out-of-range values. Missing data were handled using list-wise deletion (Kang, 2013). Basic rules of parametric analysis were followed (Polit & Beck, 2021). All analyses were conducted on the RStudio platform (RStudio Team, 2021). Scores collected from PROMIS and NIH toolbox measures were reported in alignment with HealthMeasures scoring procedures (HealthMeasures, 2025). Chi-square frequencies were used to describe the sample. A Pearson correlation test was conducted to address research question one. A score of .1, .3, and .5 indicated small, moderate, and strong correlations, respectively (Cohen, 1988).

Analysis of variance (ANOVA) tests were conducted for demographic categories with greater than three participants for each subgroup (Polit & Beck, 2021). Post-hoc tests were Levene’s Test of Homogeneity of Variance, Tukey method, and Bonferroni method in order to address research question two. For the in-group identity measure (MEIM-R), the aggregate mean score was calculated and individual summed scores were categorized as either above or below the group mean.

In order to address our third research question, multiple linear regression was used. All categorical variables were transformed and dummy coded prior to conducting tests of linear regression. We set *a priori* sample size requirements using G*power with set parameters at alpha of 0.05, 80% power, an estimated effect size of 0.25, and 5 variables (Faul et al., 2009). The minimum sample size required based on power analysis was 58, so that was our minimum target for recruitment. The mental health outcomes in the linear models were tested individually as: life satisfaction, meaning and purpose, positive affect, or anxiety.

### Model Selection Process

The model-building strategy began with PsyCap as a required predictor, given its theoretical and empirical importance. Additional demographic variables (e.g., gender, race, poverty status,) were then added one at a time. Each variable was retained only if its inclusion led to an increase in adjusted R^2^, which accounted for the number of predictors and helped to avoid overfitting. This approach ensured that the final model balanced explanatory power with simplicity, keeping only predictors that meaningfully contributed to explaining the outcome.

## Results

A total of 59 adolescents voluntarily completed the anonymous electronic survey packet. The sample was 50.8% female (40.7% male, 8.5% non-binary), mean age 15.46 [SD = 1.07 years], and 71.2% White (3.4% Black, 22% Hispanic/Latino, and 3.4% Asian). There were 37.3% that qualified for free lunch, 18.6% reported having a disability, and 3.4% reported adoption or foster history. Adolescents in this sample mostly lived with married parents (79.7%).

### Associations Between PsyCap with Perceived Rejection and PRMHOs

A Pearson’s correlation was conducted to examine associations between PsyCap, perceived rejection, and each of the patient reported mental health outcomes (PRMHOs, see Table 1). There was a moderate inverse relationship between PsyCap and perceived rejection (Pearson’s r (57) = −.36, t = −2.95, *p* = < .01). The 95% confidence interval for the correlation coefficient was [−.57, −.12]. This finding was in alignment with our theoretical expectations. There was a strong positive association between PsyCap and each of the domains of subjective wellbeing (meaning/purpose: r (57) = .53, t = 4.61, *p* = < .001; life satisfaction: r (56) = .53, t = 4.63, *p* = < .001; and positive affect: r (57) = .59, t = 5.46, *p* = < .001) and a strong inverse association with anxiety (r (57) = −.43, t = −3.59, *p* = <.001). The 95% confidence interval for the correlation coefficient between PsyCap and: meaning/purpose was [.31, .69], life satisfaction was [.31, .69], and positive affect was [.39, .74]. The 95% confidence interval for the correlation coefficient between PsyCap and anxiety was [−.62, −.19]. These findings suggest a strong positive relationship between PsyCap and domains of subjective wellbeing and a strong inverse relationship between PsyCap and anxiety.

**Table 1.** Associations Among Psychological Capital, Perceived Rejection, and Patient Reported Mental Health Outcomes

	1	2	3	4	5	6
1. PsyCap	1.0					
2. Perceived rejection	−.36**	1.0				
3. Life satisfaction	.53***	−.26*	1.0			
4. Meaning/purpose	.53***	−.15	.71***	1.0		
5. Positive affect	.59***	−.40**	.71***	.56***	1.0	
6. Anxiety	−.43***	.48***	−.30*	−.28*	−.50***	1.0

*p < .05; **p < .01; ***p < .001.

### Between Group Differences in PsyCap and Perceived Rejection

Table 2 presents the results of ANOVA tests examining how participant characteristics relate to Psychological Capital (PsyCap) and perceived rejection scores. Gender differences were significant for both outcomes. Non-binary participants reported the lowest PsyCap scores (M = 74.0 SD = 17.13) compared to female (M = 104.0, SD = 17.14) and male participants (M = 103.0, SD = 17.15), *p* = .002. We calculated the power of the one-way ANOVA test in Table 2, even with the small sample size of 5, with means 103, 104, 74, SD ≈ 17.1, sample sizes 24, 30, 5, and alpha = 0.05, the power of the one-way ANOVA was approximately 0.9 (90%). For perceived rejection, non-binary individuals had the highest scores (M = 68.7, SD = 8.92), compared to females (M = 62.2, SD = 8.93) and males (M = 58.0, SD = 8.92), *p* = .036. In-group identity was also significantly associated with PsyCap (*p* = .006). Participants who reported a strong in-group identity scored higher on PsyCap (M = 107.0, SD = 17.71) than those who reported lower in-group identity (M = 94.0, SD = 17.67). No significant differences were observed in PsyCap or perceived rejection based on race, ethnicity, poverty status, or disability status, suggesting that these characteristics did not meaningfully influence the psychological capital or perceived rejection among adolescents in this sample.

**Table 2.** Differences in PsyCap and Perceived Rejection Scores by Group

Group	n	PsyCap mean (SD)	*p*-value	Perceived rejection mean (SD)	*p*-value
Gender			.002**		.036^*^
Male	24	103.0 (17.15)		58.0 (8.92)	
Female	30	104.0 (17.14)		62.2 (8.93)	
Non-binary	5	74.0 (17.13)		68.7 (8.92)	
Race			.326		.457
Black/Hispanic/Asian	17	97.1 (18.76)		59.6 (9.32)	
White	42	102.4 (18.73)		61.6 (9.33)	
Ethnicity			.516		.554
Not hispanic/Latino	41	101.9 (18.83)		61.5 (9.35)	
Hispanic/Latino	18	98.4 (18.83)		59.9 (9.33)	
Ethnic identity			.006**		.377
Below Avg. Identity	28	94.0 (17.67)		62.1 (9.31)	
Above Avg. Identity	31	107.0 (17.71)		60.0 (9.30)	
Poverty status			.353		.258
Free lunch	17	98.4 (16.39)		62.3 (8.16)	
No free lunch	22	106.0 (21.20)		62.8 (10.55)	
Uncertain	19	97.9 (18.66)		58.2 (9.28)	
Disability			.993		.795
No	48	101.0 (18.89)		60.9 (9.35)	
Yes	11	101.0 (18.95)		61.7 (9.39)	

*Note.* *p < .05; **p < .01; ***p < 001.

Mean (Standard deviation). Avg. = average.

### Intersectional Model of PsyCap and Mental Health

Table 3 summarizes the results of multiple regression models predicting four mental health and well-being outcomes: anxiety, life satisfaction, meaning and purpose, and positive affect. The goal was to identify the most parsimonious yet explanatory model for each outcome by using Psychological Capital (PsyCap) as the core predictor and testing the added contribution of demographic characteristics.

**Table 3.** Multiple Regression Prediction Model for Health Outcomes Using PsyCap

	Anxiety		Life satisfaction		Meaning purpose		Positive affect	
	B	*SE*	B	*SE*	B	*SE*	B	*SE*
Intercep	67.83	6.55^***^	11.12	6.39	11.92	6.31	−7.70	7.69
PsyCap	−.17	.06^**^	.30	.06^***^	.28	.06^***^	.37	.07^***^
Gender								
Female	6.74	2.16^**^	−3.97	2.12			−4.33	2.57
Other	11.69	4.27^**^	3.69	4.17			−1.34	5.00
R^2^	.35		.34		.28		.38	
Adj. R^2^	.31		.31		.27		.35	

*Note.* ^*^p < .05; ^**^p < .01; ^***^p < .001.

Estimated coefficients (B) and standard error (SE).

### Key Findings

PsyCap explained 16%∼27% variances alone (not reported separately). PsyCap was a significant predictor in all models; higher PsyCap score was associated with (1) lower anxiety (B = −.17, *p* = .009, (2) greater life satisfaction (B = .30, *p* < .001), (3) more meaning and purpose (B = .28, *p* < .001), and (4) higher positive affect (B = .37, *p* < .001). For anxiety, gender was retained in the final model due to the added explanatory value. The final model yielded an adjusted R^2^ (.31), suggesting that these predictors explain a substantial proportion of anxiety variation. In the life satisfaction model, gender was included alongside PsyCap, improving the adjusted R^2^ to .31 even though it is not a significant predictor. The meaning and purpose model retained only PsyCap, as no additional demographic variable improved the adjusted R^2^ beyond what PsyCap explained alone. For positive affect, gender (female) again not significant but improved model fit with negative impact on positive affect (B = −4.33, *p* = .10).

This modeling approach highlights the central role of PsyCap in predicting psychological well-being. Additional variables such as gender and poverty status only contributed when they significantly enhanced the model’s predictive ability. See Table 3 for more details.

## Discussion

Much of what is known about psychological capital has previously been examined among adults and university students, rather than rural adolescents, a population at notably high risk for poor mental health. This preliminary study presents a novel contribution to the literature by integrating aspects of Minority Stress Theory and demonstrating through empirical evidence the utility of Psychological Capital Theory for rural adolescent mental health research. Through the analyses undertaken, we were able to answer our research questions on PsyCap, socioenvironmental characteristics (including perceived rejection and gender identity), and mental health among selected groups of rural adolescents.

PsyCap was moderately associated with both perceived rejection and all PRMHOs among this group of rural adolescents. This is the first study to date in the U.S. that explores PsyCap in the context of socioenvironmental characteristics among rural adolescents. Rural communities have many positive characteristics which make them a great environment to raise a family (Crouch et al., 2023), yet the potential for discrimination and feelings of exclusion exist, and the risk of isolation is higher for adolescents who don’t feel like they can be themselves or are worried about others’ perceptions of who they really are. This is a preliminary study, so even though we can not assume the findings are generalizable or causal, there is enough evidence to suggest a significant relationship exists and needs to be further explored. Aspects such as perceived rejection and stigma are difficult to directly impact as a healthcare provider, but inner resources such as PsyCap have been shown to be a set of characteristics that healthcare providers and school leadership may directly promote through brief, tailored interventions (Finch et al., 2023; Luthans et al., 2007), making these findings important from a clinical practice perspective to warrant future research.

We found a statistically significant difference in levels of PsyCap and perceived rejection between male, female, and non-binary adolescents. We also noted significant differences based on adolescent in-group identity. These findings build upon prior research, and highlight the need to support rural adolescents who fall outside social norms. Prior work with transgender men and women (age 16–51) demonstrated mental health disparities exist between cisgender and non-cisgender groups, and those with greater sexual fluidity had increased anxiety and decreased social functioning (Katz-Wise et al., 2018). Non-cisgender youth in Spain have been found to be at heightened risk for bullying and suicide ideation (Aparicio-Garcia et al., 2018), and transgender youth in the U.S. have had similar results (Toomey et al., 2018). The importance of social support systems to increase inclusion for at-risk groups has been previously highlighted (Lian et al., 2022; Reisner et al., 2015). The findings in our study build on this literature by providing seminal evidence of differences in PsyCap and perceived rejection among male, female, and non-binary rural U.S. adolescents, as we could find no previous research that explored PsyCap and perceived rejection between all three gender groups.

In our linear regression model, we took into account the meaningfulness of PsyCap in the presence of sociodemographic factors (gender, poverty status, and race). We could find no prior research that explored PsyCap and mental health from this health equity lens. PsyCap was a significant predictor of all PRMHOs which highlights the central role of PsyCap in predicting subjective wellbeing. A global literature review of PsyCap in relation to youth mental health found PsyCap was widely associated with positive mental and behavioral health outcomes, yet there were very few studies conducted in the U.S. and no prior studies exist which include rural adolescents (Preston et al., 2023).

Additionally, we found gender contributed to the model’s explanatory power for several of the PRMHOs (anxiety, life satisfaction, and positive affect). Gender should be considered when conceptualizing future work to promote well-being. Prior work on gender differences is inconclusive, and factors such as social norms, age-related differences, and genetics are suggested reasons for how genders’ express their well-being differently (Batz & Tay, 2018). In this study we found gender played an explanatory role in mental health, warranting the need for a deeper look into gender roles and how different gender groups report mental health and subsequent clinical outcomes.

### Implications for Clinical Practice

The benefits of motivational interviewing and brief, strengths-based goal planning interventions are well-known (Child Health and Development Institute, 2023). Curtin et al. recommends the use of strengths-based interventions tailored to the context of the social environment to decrease or prevent mental health problems among rural adolescents living on the margins (2017). Healthcare workers and educators who interact with non-binary adolescents may support their development of increased psychological capital through targeted, strengths-based interventions. As an example, collaborative, PsyCap-based goal planning (e.g., helping the adolescent develop a specific goal they want to achieve, having the adolescent create a list of ways they have contributed to the success of a project) is non-invasive, low-cost, and can be implemented in many clinical and community settings. The examples provided are introductory ideas of the possible ways to support PsyCap development among rural adolescents. In this study, higher PsyCap scores were associated with a more satisfying life, heightened meaning and purpose, and a more positive affect; all of which are important for positive mental health outcomes.

### Implications for Future Research

Our findings revealed adolescents with stronger in-group identity reported significantly higher PsyCap. We support the stance that social support, social and internal acceptance, and connectedness are protective factors for adolescents (Jones et al., 2022; Matsick et al., 2020; Preston & Rew, 2022; Resnick et al., 1993; Weinhardt et al., 2019). We recommend further investigation of the relationships among PsyCap, perceived rejection, and connectedness as an additional area for future exploration to promote the mental health of rural adolescents.

We also noted the mean scores for perceived rejection ranged from 58.0 (SD = 8.92) to 68.7 (SD = 8.92) for all groups. The general population mean for this normed scaled is 50 (SD = 10; HealthMeasures, 2025). More work needs to be done to understand why these rural adolescents report significantly higher perceived rejection than the general U.S. population, and if this finding is similar in other rural towns and/or regionally. Individual and systems-level policy should be closely examined for which strategies are currently in place to mitigate peer rejection and enhance protective factors in clinic and school settings.

### Limitations

The sample (*n* = 59) came from one rural 2-A school district in one southern state and so the results should be interpreted with extreme caution due to the small number of participants. The generalizability of this study is very limited as school, community, and cultural norms may vary across the region, state, nation, or globe. Also, this study was conducted only with English self-report measures, and there is a risk for random error in the results depending upon those who were able to and chose to complete the survey and those who were not able to or chose not to complete the survey. The data did meet all assumptions for linear regression analyses and fell along a normal distribution, which increases our confidence in the results. Furthermore, it should be noted there is the risk for Type I Error due to multiple comparisons and the risk of overfitting in the regression models. We selected the simplest model available balanced with explanatory power, retaining only those variables which increased the adjusted R^2^ value, to combat this risk.

Additionally, we want to make note that the smallest subgroup retained for ANOVA testing was the gender category for “non-binary” adolescents. We believe data for this subgroup is clinically important to retain, because although the non-binary group was an *n* of 5 (which is not in balance with the total *n* for males and females in the sample), the total sample size was 59, so the non-binary subgroup makes up 8.5% of the sample. In this region of Texas, non-binary and other gender minority adolescents are largely excluded and rejected in their small rural communities, and are at extremely high-risk of labeling, negative stereotyping, separation, power asymmetry, and in terminal cases, suicide. It is a limitation that we were unable to fully capture LGBTQ + inclusion in this study because we could only include limited gender identity items (and were unable to include sexual orientation items) in the demographic section at the request of the school district. Because many rural schools are very small (such as the one selected for the study), rural adolescents are often left out of research, although we need to learn more about the role of PsyCap in mental health for this population.

## Conclusion

The purpose of this study was met. The findings provide preliminary evidence on the utility of PsyCap Theory (and aspects of Minority Stress Theory) as a framework for rural adolescent mental health research. This study also provides preliminary evidence that psychological capital is associated with more positive mental health outcomes among rural U.S. adolescents. This study demonstrates the importance of future large-scale studies in order to make more confident assumptions regarding PsyCap and socioenvironmental factors in the broader population of rural U.S. adolescents.
